# The West African experience in establishing steering committees for better collaboration between researchers and decision-makers to increase the use of health research findings

**DOI:** 10.1186/s12961-017-0216-6

**Published:** 2017-07-12

**Authors:** Namoudou Keita, Virgil Lokossou, Abdramane Berthe, Issiaka Sombie, Ermel Johnson, Kofi Busia

**Affiliations:** 1 West African Health Organisation (WAHO), 01 BP 153 Bobo-Dioulasso 01, Burkina Faso; 2 Muraz Centre, 01 BP 390 Bobo-Dioulasso 01, Burkina Faso

**Keywords:** Steering, Information transfer, Committee for evaluation and dissemination of innovative technologies, Interprofessional collaboration, Appropriation of knowledge, West Africa

## Abstract

**Background:**

Aware of the advantages of a project steering committee (SC) in terms of influencing the development of evidence-based health policies, the West African Health Organisation (WAHO) encouraged and supported the creation of such SCs around four research projects in four countries (Burkina Faso, Nigeria, Senegal and Sierra Leone). This study was conducted to describe the process that was used to establish these committees and its findings aim to assist other stakeholders in initiating this type of process.

**Methods:**

This is a cross-sectional, qualitative study of the initiative’s four projects. In addition to a literature review and a review of the project documents, an interview guide was used to collect data from 14 members of the SCs, research teams, WAHO and the International Development Research Center. The respondents were selected with a view to reaching data saturation. The technique of thematic analysis by simple categorisation was used.

**Results:**

To set up the SCs, a research team in each country worked with health authorities to identify potential members, organise meetings with these members and sought the authorities’ approval to formalise the SCs. The SCs’ mission was to provide technical assistance to the researchers during the implementation phase and to facilitate the transfer and use of the findings. The ‘doing by learning’ approach used by each research team, combined with WAHO’s catalytic role with each country’s Ministry of Health, helped each SC manage its contextual difficulties and function effectively.

**Conclusion:**

The involvement of technical and financial partners motivated the researchers and ministries of health, who, in turn, motivated other actors to volunteer on the SCs. The ‘doing by learning’ approach made it possible to develop strategies adapted to each context to create, facilitate and operate each SC and manage its difficulties. To reproduce such an experience, a strong understanding of the local context and the involvement of strong partners are required.

## Background

The scientific literature has highlighted the role played by steering committees (SCs) [1–5], monitoring committees and advisory boards in health research [6–13] and healthcare [14, 15]. A SC is a group of actors (leaders from among project stakeholders) who meet on a regular basis to decide on, guide and evaluate a project’s implementation, and to recommend strategies on how to best achieve the project’s goal and objectives. In a research programme, SCs can play key roles in the development of research, the transfer of research findings to potential users (community, practitioners and decision-makers) and the appropriation/use of findings by users. Lemire et al. [16] have described the approaches, steps and determinants of the knowledge transfer process. The transfer or use of scientific knowledge cannot take place without direct or indirect interaction between researchers, actors/beneficiaries and decision-makers. Close, continuous collaboration facilitates or guarantees the success of the transfer and/or production and use of findings. To achieve this, bringing stakeholders together in a functional SC, in turn, supports the establishment of dynamic collaboration. SCs have become indicators of strong involvement in research by non-researcher actors, which is a good strategy for establishing multiactor, multisectoral and multidimensional partnerships. Such partnerships allow the various parties’ needs, aspirations and resources to be jointly taken into consideration [13]. Through the scientific literature [5], these different parties and their technical and financial partnerships have become well aware of the advantages of these committees. However, they are not aware or do not have a good understanding of how these committees are established, given that the same scientific literature rarely documents the process for setting them up. According to Uneke et al. [5], a health policy advisory committee is a mechanism that can serve as an excellent platform for interaction between decision-makers and researchers. The authors demonstrated that the establishment of a health policy advisory committee can stimulate efforts by ministries of health to apply evidence-based strategies to improve their services. However, these authors, like many others who discuss SCs, have not described how to establish these types of committees. This article helps make up for the lack of scientific documentation on the process for establishing SCs around a health research project.

In West Africa, knowing the advantages of these committees, the West African Health Organisation (WAHO) promoted and supported their creation during the West Africa Initiative to Strengthen Capacities through Health Systems Research. This initiative aimed to strengthen the capacities of researchers, actors and decision-makers in health system research, and in the transfer and use of health system research findings. To design this project, WAHO started from the premise that, in West Africa, many health indicators are low. Although numerous health research findings exist, actors and decision-makers make little use of them [17–19] due to limited access to research findings, difficulties understanding the findings, actors doubting or not accepting the findings, findings that are contrary to the ways of thinking, acting or being of non-researcher actors and researchers, and lack of involvement by non-researcher actors in the process of generating findings, among others [5, 17–20]. The different actors in health systems research (researchers, practitioners, decision-makers) are not mutually acquainted and do not collaborate much, by profile or between profiles, by country or between countries. Good practices in research and/or health interventions are not visible or widely shared. A culture of policies and practices based on scientific evidence is also lacking [5, 17–21], particularly with regard to feedback. This initiative is therefore WAHO’s contribution to boosting, promoting and strengthening collaboration between researchers, actors and decision-makers through the establishment of health research project SCs to increase the use of findings and improve equity and governance in health systems. WAHO received financial support from the International Development Research Center (IDRC).

At this stage of the initiative, the research teams are in the process of analysing the findings of their research. The SCs will utilise these findings to influence decision-making in health policies and programmes. It is therefore difficult to make a statement about the effectiveness of these SCs. A specific article will analyse the effectiveness of these SCs 1 year after the initiative. However, a study on how these SCs are established is possible at this stage of the initiative’s implementation. Therefore, the objective of this article is to describe and analyse the process that was used to establish SCs around four research projects under the West Africa Initiative to Strengthen Capacities through Health Systems Research to help other actors who wish to undertake this type of collaborative tool.

## Methods

This was a qualitative, descriptive, analytical study conducted between February and April 2016 on the SCs or monitoring committees of four projects under the West Africa Initiative to Strengthen Capacities through Health Systems Research. This initiative was implemented by consortia of research or intervention organisations in Burkina Faso, Nigeria, Senegal and Sierra Leone. The consortia were made up of the research team and the organisations that the SC members came from. These consortia addressed the topics of the development of a process for assessing the performance of the district health system (Burkina Faso); strengthening of the health system through better, equal access to primary healthcare (Nigeria); funding, equity and governance in the health system (Senegal); and barriers faced by pregnant women to free access to health institutions (Sierra Leone).

Our first step in this study was to conduct a literature review by exploring various databases (primarily PubMed and Cairn) using different keywords (Advisory Committees, Professional Staff Committees, Committee Membership; Steering; Information Transfer; Committee for Evaluation and Dissemination of Innovative Technologies; Interprofessional Collaboration; Appropriation of Knowledge). We also explored various documents regarding the SCs of these four projects. We used this review to build a better interview guide.

The target population for the collection of qualitative data was made up of members of the SCs (referred to as a ‘monitoring committee’ in Burkina Faso), researchers from the research teams, and WAHO and IDRC staff involved in this initiative. Within this population, respondents were selected in a reasoned and expedient manner. We capitalised on the peer review workshop (meeting between the different research and intervention consortia from the four countries) in Dakar, Senegal, in February 2016 to collect data from participants. At least two people (one researcher and one SC member) were surveyed per country. This most often meant the lead researcher and/or their representative and/or a researcher, and the SC chairperson or their representative and/or an SC member. Interviews were conducted with each respondent(s) of a country until data saturation was reached. Saturation was reached with a respondent when we felt we had obtained all the information needed to satisfy the study objective and continuing the interview would not yield any new strategic information. However, over the course of the analysis, email exchanges still took place with respondents to obtain further strategic information.

The guide was administered to each respondent by the same researcher/interviewer. This researcher/interviewer was an independent consultant who was not a member of either WAHO or IDRC, nor a member of one of the research teams. However, this person was familiar with this West African initiative. The guide was developed around the following points: introduction of the respondent, the process used to create the SC, the method used to establish the SC, the SC’s composition, how the SC operates, the SC’s resources and mission, strengths and weaknesses of the process, and difficulties encountered, among others. Interviews were recorded and transcribed in French. Although anonymity was maintained, the respondents were aware that the resulting study report and article would be accessible to all actors in the initiative. It is possible that this situation prompted some respondents to use ‘politically correct’ speech so as not to harm any partners or stakeholders.

This study is characterised by its comprehensive approach [22], which seeks to understand the respondents and the study topic. This approach placed a great deal of importance on the respondents’ statements, motivations (underlying drivers of an action or decision) and rationalities. It considered the context in which the four projects are being carried out. The study also used the systematic approach [22], which favoured the principle of circular causation and plural truths. From its design through to its valorisation, this study adhered to the ethical principles of scientific research in health. The position of ethical relativism [22] in qualitative research was adopted throughout this study. Finally, this study used grounded theory on the data [23]. No initial hypothesis was formulated. However, during the literature review, the researchers were particularly interested in the findings or explanatory models of other authors [1–4, 7, 9, 12, 13].

The data from this study was manually scrutinised as it was collected. The technique of thematic analysis by simple categorisation was used [22]. The selected categories included the SC’s creation, opportunities encountered during creation, difficulties encountered, the SC’s composition, how the SC operates, and the SC resources and mission. Each major finding was interpreted (to give it meaning within its context) and then strengthened or rebutted by the writings of other authors.

## Results

In total, 14 people, including four SC members, seven researchers and three members of sub-regional (WAHO) or international (IDRC) organisations participated in this study. All participants had been involved in the initiative since its inception.

### Origin of the idea of establishing SCs

According to some respondents, the idea of establishing the SCs was implicitly alluded to in the competitive call for concept notes launched by the IDRC and WAHO in August 2012. This call invited multipartite teams of researchers, decision-makers and practitioners to present concept notes on two eligible subjects, namely equitable access to health systems, and health system governance and governance structures. Moreover, Specific Objective 2 of this call specified that the initiative was aimed at strengthening ties between multipartite groups of researchers, practitioners and decision-makers charged with tackling specific issues that they had identified in advance. To be eligible for this call, one of the criteria was that the application had to be presented on behalf of a multipartite consortium consisting of researchers, decision-makers and practitioners able to define a problem with respect to strengthening health systems and use the research findings to begin to tackle that problem.

Finally, the call specified that applicant teams had to consist of both researchers and partners and that a team’s composition represented 20% of the selection criteria. These factors therefore led to the idea of establishing an SC in most of the consortia that responded to this call:

“*From the project’s inception, we* [Senegal’s Ministry of Health and Social Action] *were asked to first lend the ministry’s political support, to show that this project, by way of its formulation, could be of interest to the Ministry of Health. So we wrote a letter of support for the application.*” (Member 1, Senegal SC)

Thus, the researchers identified the first partner organisations that could or should be involved in the SCs (potential partners) to establish their consortium and respond to the call for concept notes. Around 60 West African consortia prepared concept notes in response to this call. Following a review process, seven proposals were preselected based on strict technical criteria, namely relevance and potential impact of the research project (40%), relevance and potential scientific merit (40%), and composition of the teams and partnerships (20%).

To help the consortia better prepare their final submissions for review, a protocol development workshop was held in Bobo-Dioulasso, at WAHO headquarters, in October 2012. During this workshop, the preselected consortia interacted with specialists from the Regional Advisory Committee established by WAHO and IDRC. This time, the idea of establishing SCs was explicitly suggested to the consortia:

“*From the start of the process, at a regional dialogue meeting organized in Dakar by IDRC in November 2011, when all participants in this dialogue chose WAHO as the supranational institution to make a proposal, WAHO’s main proposal was to implement SCs around teams. This idea was well received by IDRC. During the meeting with the seven preselected teams in Bobo-Dioulasso, WAHO specifically included the SC option. Beyond the research questions, research design, methodological approach, and so on, the teams were asked to take the SC’s establishment into account and, therefore, be prepared to implement the SC upon their return and involve them in the finalization of the research questions discussed in Bobo-Dioulasso.*” (Interview with a WAHO official)

This information was confirmed by all the respondents, who unanimously stated that the idea of establishing SCs that bring together researchers, health practitioners and decision-makers came implicitly and explicitly from WAHO. By promoting and supporting the creation of these SCs, WAHO was working from the premise that gathering researchers, actors/stakeholders and decision-makers within a functional framework of regular meetings would have numerous consequences. It allowed them to co-define a highly practical research topic based on the needs and aspirations of actors and decision-makers, while considering the resources, skills and capacities of researchers. It promoted the co-production of evidence-based findings and the communication of these findings. It allowed the mutual appropriation of these findings so they could be used or converted into action to benefit a community or the general public.

All the researchers interviewed recognised that the idea of establishing these SCs was explicitly expressed during that meeting. According to one researcher, the idea of establishing the SCs seemed “*natural*” in this initiative, because everything (the call for concept notes, the recommendations from the Regional Advisory Committee specialists, the researchers’ experience) was pointing to these SCs being indispensable:

“*The idea of setting up an SC arises out of several concerns. First, the call for applications clearly stipulated that applicants must clearly show how they planned to achieve a certain degree of appropriation and use of their research findings… But beyond all of that, it was also a recurring concern. In general, at the ministry here* [Burkina Faso]*, it is fairly systematic when we want to carry out these types of activities. Ministry actors are closely involved from the beginning. What we typically do is set up an SC or a monitoring committee.*” (Member of the Burkina Faso research team)

After the meeting with the specialists, four consortia were selected. These consortia participated in a research protocol development workshop in Dakar in May 2013, and each one met again with its country’s Ministry of Health, an organisation that had to be involved in the consortium to have the chance of being selected definitively.

### Establishment of the SCs

In each country, the SC’s establishment truly began after the definitive selection of the consortium. Once the researchers, namely the leaders of these consortia, were certain that they had been selected, they began by notifying other potential partners of their consortium. Each lead researcher established a list of potential SC members and met with the country’s Ministry of Health to discuss the SC’s establishment. In general, in addition to email and telephone exchanges, two or three informal meetings were needed to obtain the quasi-definitive list of SC members and organisations. Based on the data collection area and the topic addressed, the researchers identified the organisations most affected by their research and its findings. This list was amended by the Ministry of Health to obtain the quasi-definitive list. In each selected organisation, the top official designated their representative, and the choice was left to the top official’s discretion. In most of the four countries, a document from the Ministry of Health or local health authority formalised the SC’s creation. Each SC was linked to the Ministry of Health via the organisation in charge of the said Ministry’s research; this organisation was SC chair.

Once the SCs were established, a first official meeting was organised to give members an opportunity to get better acquainted, introduce themselves and amend the research protocol. During this meeting, the SC was officially launched via a statement made before the health authorities.

### Factors that facilitated the SCs’ establishment

According to the respondents, there were certain facts, actions or situations that facilitated the SCs’ creation. Respondents unanimously cited the commitment, determination and support of WAHO, which, as a supranational organisation, directly asked each country’s Ministry of Health to get involved at all stages of the project in their country (the SC’s creation, design, implementation, valorisation, transfer and use of findings). This was confirmed by a WAHO official:

“*To encourage the SCs creation, the WAHO Director General sent a letter to each Ministry of Health concerned. In the letter, the Minister of Health was asked to make their skilled services available to work with the research team. WAHO introduced these teams and their research topic and asked for the ministry’s commitment to facilitate all the research and the use of the findings. WAHO also travelled to each country to support them in establishing the SCs.*” (WAHO official)

In each country, the actors recognised that the commitment of the Ministry of Health on a national (Burkina Faso and Senegal) or local (Nigeria and Sierra Leone) level and the Ministry’s experience in establishing SCs for similar projects also facilitated the SCs’ creation:

“*The Ministry of Health and Social Action is accustomed to establishing SCs. In general, when it wants to set up an SC, it tries to take all actors into account from the beginning and involve them as much as possible in the activities. This generally includes everyone: Ministry of Finance, the Assembly, the economic council, the environmental, schools, the university, society, and so on. The ministry really tries to cast a wide net. And the ministry is accustomed to doing so.*” (Member 2, Senegal SC)

According to the respondents, in addition to these commitments (supranational and national), in each country, the lead researcher was invested at several levels (telephone or Skype calls, sending emails, informal meetings).

Aside from these multifaceted investments, collaboration experiences between the lead researchers and the Ministry of Health, their contacts or the extent of their relationships within the Ministry of Health greatly facilitated the SC’s creation. Finally, positive perceptions of the teams, the research topic and the logic/design of the SC facilitated the non-financial motivation and support of potential SC members, who agreed to be SC members on a volunteer basis.

### Size and composition of the SCs

The size of the SCs (number of members per SC, including at least two researchers) varied from one country to another. There were 10 members in Burkina Faso, 18 in Sierra Leone SC, 20 in Senegal and 23 in Nigeria. According to the respondents, there was no quota set for men/women per country on the SCs, but in each country, at least one-fifth of the members were women. The actors opted more for representation by institution or department than by sex. Each institution/department enrolled in the SC was free to choose the right person from within its ranks to be on the SC. In each country, the SC’s official charter specified that, based on the activities, the SC members could enlist other members or organisations. From the SCs’ establishment to the time of the survey, the number of members per SC had not changed. However, due to occupational mobility in the positions or institutions, the individuals comprising the SCs often changed. In these cases, these individuals were replaced systematically, without this affecting the functioning of the SC. For example, this was the case in Senegal with the death of the WAHO Focal Point, and in Sierra Leone with the transfer of the Bombali District hospital’s chief physician.

As for the SCs’ composition, each country established multisector SCs. This composite make-up was in line with one of WAHO’s strong recommendations:

“*WAHO insisted on the idea of setting up an SC, making it as heterogeneous as possible, and that the members be actors affected by the study, with the possibility that these actors could even begin using the intermediate findings prior to the end of the research project.*” (Interview with a WAHO official)

In all four countries, members came from the following sectors: parliament (local representatives), communications and information (journalist), health (administrative and operational health officers), transportation, teaching and education, defence and security (police, gendarme), and civil society (health protection and promotion association). The SCs of Sierra Leone, Nigeria and Senegal, composed of more than 10 people, were more heterogeneous than the SC of Burkina Faso (10 members), which was essentially, even exclusively, made up of members from the health sector. The fact that this study is strictly limited to the process of establishing SCs does not allow us, at this stage, to determine the impact of each SC’s composition choices.

### Roles/missions of the SCs

Overall, the SCs took on roles or missions in three areas. First, to facilitate research, the SCs had the mission of validating, amending/adjusting research projects, supporting researchers in the implementation of their research, and external validation of the research findings. Second, the SCs also had the mission of facilitating the transfer of research findings to their potential users. Finally, the role of SCs was to facilitate the appropriation and use of research findings.

### SC operations

For operational purposes, the SCs’ creation was formalised by ministerial order (Burkina Faso and Senegal) or formal terms of reference (Nigeria and Sierra Leone). WAHO stated that it did not insist too much on the SCs’ formalisation because the entire West Africa Initiative to Strengthen Capacities through Health Systems Research is based on the ‘doing by learning’ strategy. For the countries, this meant establishing their SCs by adopting a less specific, non-pre-established process of doing to set up their SCs and learning lessons over the course of this action. Too much formalisation would therefore stifle learning:

“*It is truly an initiative that is based on learning by doing. Each country had to set up its SC based on its own context. WAHO did not want to formalize, or impose, a standard format on the countries. Moreover, overly administrative formalization could make the researchers back away. Instead, the researchers had to be encouraged to work, to approach all the stakeholders in order to set up the SCs.*” (Interview with a WAHO official)

The SCs operated with financial resources held by the IDRC-funded research teams. Material resources came from the research teams or from the SC chairs’ organisations. SC meetings were led by their chairperson. Within all the SCs, decisions were made by consensus. Members never relied on voting to decide. There was no established quorum to be reached to validate the SC meetings. In practice, members attended the various meetings most of the time. The SCs operated on a volunteer basis; over the course of the meetings only the members’ transportation or fuel was reimbursed. This was agreed to by the members, who, for the most part, were accustomed to this way of doing things with other partners. The promoters of these SCs (WAHO, researchers and the Ministry of Health) justified this choice out of their common concern for ensuring the SCs’ sustainability/systematisation in the most cost-effective manner, considering the scarcity of financial resources. There was no formal or direct link between the four SCs. However, annual meetings of the initiative’s consortia provided opportunities for meetings between the research teams, SC chairpersons, and WAHO and IDRC specialists/experts. These meetings allowed for the sharing of experiences and the strengthening of collaborations between actors within a country and from one country to another. WAHO oversaw the monitoring and regional coordination of the initiative.

Each SC planned two meetings a year. In 3 years, the actual number of SC meetings varied from three in Senegal, to four in Sierra Leone, and to five in Burkina Faso and Nigeria. For one meeting, the researchers took over for the SC chairperson, submitting the draft agenda to him. After amending it, the chairperson officially convened the other members of the SC.

### Assessment of the SCs’ impact

The respondents had a positive impression of the SCs in terms of composition, institutional anchoring and operations. In each country, the SC’s anchoring to the Ministry of Health was perceived as a strength that enabled it to fully play its role in the three areas (facilitation of research, transfer of research findings and the use of these findings). All three parties involved (researchers, actors/stakeholders and decision-makers) were happy to be on an SC that would make the co-production and co-utilisation of research findings possible. The establishment of these SCs in the different countries met an implicit expectation of the parties. In Nigeria, for example, the federal Ministry of Health made an official request to WAHO to better understand and become more involved in the programme. Formal ties were established between the Delta State Ministry of Health (which runs the SC) and Nigeria’s Federal Ministry of Health. These ties should provide for better co-production of policy briefs and facilitate their appropriation and use. One indicator of the SC’s acceptance by national health authorities is having the SC members appointed/designated by way of an official document and agreeing to have one of the specialised organisations head the SC. On the other hand, the respondents regretted the lack of financial autonomy of the SCs, which relied heavily on the researchers, without whom the SCs could not meet. In Sierra Leone, the SC operations were disrupted for a long time (from May 2014 to November 2015) by the Ebola virus epidemic. Some respondents lamented the fact that the SC members’ skills were not strengthened in terms of facilitation and operations, support for research, and transfer and use of research findings. However, according to the respondents, the various regional meetings and support visits from the WAHO team and from technical experts in the countries provided opportunities to share ideas on potential approaches to facilitation, support for research, and transfer and use of research findings by the SC members present.

### Actors’ roles in the SCs’ establishment and operations

In this West African initiative, IDRC supported WAHO financially. IDRC also directly supported the country research teams in conducting their research for practical purposes. IDRC approved WAHO’s initiative and its SC development strategy through the ‘doing by learning’ principle. Financial support from IDRC was a way of helping WAHO explore the SCs’ value and their capacity to influence health policies and practices in West Africa. WAHO played a managerial (sub-regional interface) and technical role. Due to its status as a supranational organisation, it encouraged the research teams to go to their Ministry of Health, and it asked the ministries to support its researchers and facilitate the SC’s establishment, operations and achievements. WAHO also oversaw the SCs’ activities, partially from a distance and partially up close. The mission of the Ministries of Health was to facilitate the SCs’ establishment and operation. Except for the reimbursement of the SC members’ fuel expenses and lunch expenses during meetings (which were covered by the researchers on IDRC funding), the ministries hosted the SC meetings, providing meeting rooms and covering related costs (water, power, maintenance costs, audio-visual equipment). The role of the researchers was to establish the SCs in close collaboration with the Ministry of Health, facilitate the organisation of regular SC meetings, conduct research in close collaboration with other SC members, regularly report on the progress of their research and/or regularly share their findings with other SC members. The role of the other SC members was to participate regularly in SC meetings, guide the entire research process to produce highly practical data, promote and facilitate knowledge transfer, appropriate this knowledge and use it to improve the health of the target populations.

### Difficulties encountered in the SCs’ establishment and/or operation

The respondents from Nigeria reported that there were no major events that significantly disrupted the establishment of their SC. In Burkina Faso, the team of researchers cited administrative formalities and staff mobility issues as slowing down the SC establishment process.

Additionally, given that they were conducting research in two regions of Burkina Faso, Burkinabe researchers attempted to set up one SC and two regional committees. After a year of wavering, they realised that it would be difficult for these regional committees to function due to the project’s limited financial resources. However, the research team used official and informal means of communication to collaborate with health officials from the two regions to collect data and hold the planned deliberative workshops.

In Sierra Leone, the respondents acknowledged that they hesitated for some time on the geographic location of their SC. For greater effectiveness and efficiency, they debated whether it should be in Freetown or in Makeni (capital of the Bombali district where most of the research took place). In the end, the SC was set up in Makeni, where the research team and local stakeholders are based. The research team and WAHO then had to maintain regular contact with national officials at the Ministry of Health, which was made possible through frequent WAHO visits in Sierra Leone and the research team’s formal and informal meetings with the Ministry of Health. Another hesitation was regarding the role or mission to be allocated to the SC. Should the SC conduct the research or support the researchers in implementing the research? In this case, this was mainly due to the enthusiasm of certain SC members who wanted to participate directly in the field data collection process. Both the research team and WAHO, during support visits, could provide clarifications on this issue. The Sierra Leone consortium perceives these two forms of hesitation as major difficulties that delayed the establishment of their SC.

In Senegal, given that the research was conducted by research professors from the Université Cheick Anta Diop, and therefore under the auspices of the Ministry of Higher Education, the actors hesitated about the SC’s institutional backing – should it be anchored to that ministry or to the Ministry of Health and Social Action? In response to this dilemma, the Senegalese research team first attached its SC to the Ministry of Higher Education. Learning quickly from its mistakes, it then attached itself to the Ministry of Health and Social Action:

“*The initial actions were carried out by Higher Education. This did not facilitate the mobilization of this committee; people thought it was a university matter.*” (Member 1, Senegal SC)

WAHO sent the Senegalese Ministry of Health an official correspondence and copied the research team on that correspondence, ultimately facilitating the team’s decision to collaborate mainly with the Ministry of Health. In addition, it should be noted that, beyond the formal meetings, there were regular telephone and email exchanges between the research team and certain contact persons from the Ministry of Health, particularly to obtain certain evaluation documents or financial studies that were already available at the ministry.

## Discussion

### Origin of the idea of establishing SCs

It is obvious that, in the four countries of this West Africa initiative, the idea of establishing SCs was implicitly and explicitly suggested by WAHO. Therefore, it was this organisation that promoted and supported the SCs’ operations. Various factors explain this involvement of technical and financial partners in the promotion and support of the SCs’ operation. In this and other contexts, researchers, decision-makers and practitioners often lack a professional culture that is conducive to the creation of quasi-permanent concerted action frameworks for co-decision and co-action, but it is also often the case that there is an objective or subjective lack of financial resources to organise a minimum number of productive meetings. The involvement of a technical and financial partner has the advantage of providing these resources, but the potential disadvantage is the building of unsustainable SCs that operate only to satisfy this partner.

The scientific literature [1, 5, 8, 13] shows that different actors (the community, authorities, researchers, or technical and financial partners) can collectively, or through concerted action with others, initiate the establishment of a committee. A committee’s appropriation, autonomy and sustainability come more easily if the idea or need to establish it and the financial resources for it come from the community, beneficiaries or authorities. It becomes more difficult when these come from researchers and/or their technical and financial partners. In the latter case, the committee’s existence is perceived as a ‘researchers’ thing’ and is very often tied to the existence of the research project [1, 8, 13]. Other non-researcher actors take more time to appropriate it and to mobilise the resources required for its sustainability. In the context of this initiative, the volunteer nature of the work [24, 25], and the reduction of the SCs’ operating costs, should facilitate their appropriation and sustainability by the ministries of health concerned and even help to export the model to the 11 other member nations of the Economic Community of West African States (ECOWAS). One of the originalities of this SC model is not the SC as such, since, to varying degrees, each country had its positive and negative experiences with the SCs. The originality lies more in the involvement of a supranational organisation (WAHO) financially supported by an international organisation (IDRC) and the role played by the former.

### Establishment of the SCs

This study shows that the availability or the guarantee of availability of financial resources is a leverage for the process of establishing an SC. Without a minimum of financial resources, if only for coffee or lunch breaks at SC member meetings, it is difficult to attract volunteers for the committees. To establish a committee and make it operational, a minimum of financial resources is therefore required. Moreover, in a committee, the one holding the financial resources is also the one holding and/or monopolising the strategic issues. In this West African experience, WAHO and IDRC – as the technical and financial partners of each country’s consortium – had a certain power (of mobilisation, direction) over the researchers, who also had power over the rest of the consortium’s actors. Therefore, the researchers, as the ones holding the financial resources, were the architects and leaders in the establishment and operation of the four committees. Even though they did not hold the position of SC chairperson in each country, the fact that the researchers held the financial resources meant that they were the SCs’ invisible chairpersons. Within the SCs, nothing strategic could be decided without their consent. The SC chairs set the date, time and agenda of the SC meetings based on the researchers’ suggestions. Therefore, it was the other non-researcher actors who marched to the beat of the researchers’ drum, not the other way around. This way of doing things has advantages and disadvantages, but these will not be properly documented until after the initiative’s overall evaluation is complete. The researchers’ leadership role was granted to them by WAHO and IDRC through the call for concept notes and through their funding of the committees. The granting of this role illustrates the confidence that WAHO and IDRC had in the researchers, which undoubtedly impacted the quality of their involvement.

Furthermore, the granting of this leadership role to the researchers also illustrates WAHO’s vision, namely that the SCs should ultimately be established by the researchers. We are therefore in a logic of research (first) and action (later).

These SCs were also set up through the involvement and determination of WAHO. This supranational organisation, by virtue of its status and missions, has political, technical and financial power over the ministries of health (taken individually) of ECOWAS member countries. Its involvement strengthens the credibility of consortia that defend both national and regional causes. The involvement of a strong partner such as WAHO facilitates the establishment of committees insofar as each member organisation of a country’s consortium is generally limited to finding actors who are willing and available to fulfil the role that is expected of them. However, in reality, researchers and/or actors often need multifaceted support (informational, emotional, material, technical or financial) from their partner to initiate, operate and sustain their SC or advisory committee [13]. To establish the SCs in each country, the researchers drew heavily on their experience and knowledge of the field.

The double selection (pre-selection from concept notes and protocol-based selection) allowed WAHO and IDRC to choose the best candidates. The scientific literature [7, 9, 12, 13] shows that, to establish an SC or advisory committee, when the researchers are unfamiliar with the field, they do a quick qualitative study using the participative or classic method to identify potential members of their committee. In the context of this West African initiative, the research teams’ experience and the involvement of each country’s Ministry of Health meant that this approach was not necessary.

### Factors that facilitated the SCs’ establishment

In addition to WAHO’s involvement, the involvement of each country’s Ministry of Health also facilitated the set up and operation of the committees. This initiative’s potential benefits to these ministries explain their commitment. Moreover, the experiences of ministries and the organisations comprising the committee also facilitated its establishment.

In general, the more naive the organisations or individuals are about something new, the more reluctant they may be to get involved. Experience with similar initiatives strengthens self-confidence to get involved with a new one. Anchoring the SCs to the ministries of health (public and quasi-permanent organisations) also allowed the SCs to be operational, and it facilitated their appropriation and probably their sustainability by health policy authorities.

Certain factors associated with the lead researchers (history of collaboration with potential committee members, degree of penetration within the Ministry of Health) also greatly facilitated the SCs’ creation. We reaffirm that, through their consortium pre-selection and selection process, WAHO and IDRC gave themselves a double opportunity to secure consortia with the most experienced lead researchers who were motivated to achieve the initiative’s objectives. Additionally, after having applied twice to be chosen, the lead researchers felt ‘responsible’ for the successes and failures of the country project, which explains their great determination. Finally, the enthusiasm and motivation of potential members who became actual committee members also facilitated the SCs’ establishment and operation.

They were motived by the commitment of the researchers, health authorities, the issue being tackled and the committee design. Often, with or without conviction, subordinates passively or actively go along with initiatives that are already supported by their superior because they believe or do not want to destroy the quality of their relationship with their employer. In the context of this West African experience, it was essentially the SC members’ motivation that led them to participate as volunteers.

In summary, a non-financial motivation system mobilised all country actors, as well as WAHO, in the committees’ establishment and facilitation. Systematically, the motivation of each actor/member drove and strengthened the motivation of the other actors. In other words, the motivation of IDRC motivated WAHO. This, in turn, strengthened IDRC’s motivation. The motivation of the technical and financial partners motivated the researchers, ministries of health and other actors. Their motivation, in turn, reinforced the technical and financial partners’ motivation. This motivation system is a key aspect that fostered the creation and operation of each SC with volunteer members.

The ‘doing by learning’ strategy, or flexibility in the approach to the SCs’ establishment, was also beneficial, allowing for the adoption of an approach adapted to the contexts, strengths and obstacles of each country.

### Impact assessment

According to the respondents, the strengths of this SC establishment process lie in its composition, its institutional anchoring and its operation. This has already been highlighted in the scientific literature [1, 3, 4, 6, 8, 10, 13, 24, 26–28], which adds that interpersonal, organisational, human and financial factors influence the SCs’ success and that the SCs’ institutional anchoring determines their weight or capacity to influence policies. In the context of this West African initiative, the SCs’ size and the members’ profiles varied by country. While encouraging them to establish SCs, in keeping with its ‘doing by learning’ logic, WAHO left it up to the countries to freely establish their SC. In terms of committee establishment, there is no set rule regarding the size or specifying its composition [18]. The creation of an SC is like an artistic creation, where nothing is done at random, nor is it done according to a pre-established rule. The approach for establishing SCs is therefore systematic; in other words, it is circular (back-and-forth actions are possible during the process) and adaptable to the socio-political and organisational context, the topic explored, and the needs and aspirations of potential SC members in each country. The approach is also comprehensive, aware of the potential actors/members and the context. The flexibility/adaptability of the approach also facilitated the SCs’ creation and operation. This is why the SCs’ composition varied from one country to another, depending on the context. For example, to conduct research on the barriers faced by pregnant women to free access to health institutions, Sierra Leone established a committee consisting of officials from the transportation, defence and security sectors.

The themes or social facts studied by the consortia involved multiple sectors; therefore, the countries also established heterogeneous, multisector SCs to obtain concerted, multisector responses. It is true that the four SCs had no direct link between each other. However, there were exchanges by affinity. Furthermore, the fact that the four countries’ SCs adopted nearly the same mission is proof that the country consortia communicated with one another directly and/or through WAHO, which provided a form of supervision over the initiative’s work.

Two major facts can be considered weaknesses of the process, namely the lack of financial autonomy of the SCs, which were closely dependent on the researchers; and the lack of SC member training in the facilitation and operation of the SCs, research support, and the transfer and use of research findings. Granting leadership of the SCs’ to the researchers was the desire of WAHO, which wanted to test this model and learn from it. The upcoming report on the SCs will reveal the lessons learned.

### Actors’ roles in the SCs’ establishment and operations

Although IDRC limited itself to providing financial support to WAHO and the research teams/consortia, most actors/researchers from this initiative perceive it as being the sponsor of the research developed in each country. Many things were implemented by considering the timeframes and indicators validated by IDRC. Within some research teams, this project is distinguished from other projects by the IDRC project acronym. Sometimes things are carried out as though the researchers were seeking to satisfy IDRC first, then WAHO, and finally the Ministry of Health. In other words, the researchers thought that their accountability to the financial partner and technical partner was greater than to the collaborating or beneficiary partners. This situation or the researchers’ sense of accountability to the financial partners is understandable, given the context of the scarcity of resources allocated to research. The one who holds the financial resources is systematically the one who controls the research issues.

In this initiative, WAHO’s power was reduced by the fact that it was not the organisation authorising the funds for each country’s researchers/consortium. The fact that they obtained financial resources directly from IDRC and had to justify these funds to that organisation made them feel more accountable to IDRC than to WAHO.

The different countries’ ministries of health were content to play the role delegated to them by the technical and financial partners and researchers. No ministry made an individual contribution in terms of financial resources to develop the initiative beyond the resources granted by IDRC.

### Difficulties encountered in the SCs’ establishment

Unlike the factors that facilitated the committees’ establishment (factors that were common to the four countries), the factors or situations that made their establishment difficult were country specific. Apart from Nigeria, the countries reported difficulties such as administrative formalities and staff mobility (Burkina Faso), and hesitation regarding institutional anchoring and/or the SC’s missions (Senegal and Sierra Leone). Issues about belonging to an SC and/or the control of said committee explain these difficulties.

While being members of the same consortium or committee, the actors, whether rational or strategic [29], develop techniques of varying degrees of effectiveness to control the committee and increase their power and their gains. This led to hesitation, power struggles or increased complexity of procedures. In this type of situation, officially, each actor tries to put their best foot forward and help the group establish the best committee. In practice, according to Cinq-Mars [3], various sources of influence, including the protection and strengthening of one’s discipline/institution, defence of one’s interests and preference for a particular theoretical model (that helps make their sectoral and institutional identity the cornerstone), determine the quality of the collaboration.

## Conclusion

The West Africa Initiative to Strengthen Capacities through Health Systems Research conceived by WAHO allowed heterogeneous SCs to be established in Burkina Faso, Nigeria, Senegal and Sierra Leone. This required the involvement of WAHO as a supranational structure, the leadership of researchers, the commitment of health authorities and committee members, and a non-financial motivation system. The involvement of technical and financial partners motivated the researchers and ministries of health, who, in turn, motivated other actors to participate in the SCs on a volunteer basis. The ‘doing by learning’ approach made it possible to develop strategies adapted to each context to create, facilitate and operate each SC and manage its difficulties. Individual factors (such as leadership by the researchers), collective factors (such as the reciprocal motivation system), intra-country factors (commitment and motivation of the health ministries) and supranational factors (political and technical support of WAHO, technical and financial support of IRDC) contributed greatly to the success of the SC establishment process. This experience shows that, when the technical and financial partners of health policy authorities, researchers and non-researcher actors come together to stimulate and support the creation and operation of SCs, these committees have a greater chance of success. To reproduce such an experience, a strong understanding of the local context and the involvement of strong, motivated partners are required.

## Abbreviations

ECOWAS: Economic Community of West African States
IDRC: International Development Research Centre
SC: steering committee
WAHO: West African Health Organisation
